# NG2‐glia crosstalk with microglia in health and disease

**DOI:** 10.1111/cns.13948

**Published:** 2022-08-23

**Authors:** Zuo Zhang, Xiaolong Li, Hongli Zhou, Jiyin Zhou

**Affiliations:** ^1^ National Drug Clinical Trial Institution, Second Affiliated Hospital Army Medical University Chongqing China

**Keywords:** crosstalk, health, microglia, NG2‐glia, sickness

## Abstract

Neurodegenerative diseases are increasingly becoming a global problem. However, the pathological mechanisms underlying neurodegenerative diseases are not fully understood. NG2‐glia abnormalities and microglia activation are involved in the development and/or progression of neurodegenerative disorders, such as multiple sclerosis, Alzheimer's disease, Parkinson's disease, and cerebrovascular diseases. In this review, we summarize the present understanding of the interaction between NG2‐glia and microglia in physiological and pathological states and discuss unsolved questions concerning their fate and potential fate. First, we introduce the NG2‐glia and microglia in health and disease. Second, we formulate the interaction between NG2‐glia and microglia. NG2‐glia proliferation, migration, differentiation, and apoptosis are influenced by factors released from the microglia. On the other hand, NG2‐glia also regulate microglia actions. We conclude that NG2‐glia and microglia are important immunomodulatory cells in the brain. Understanding the interaction between NG2‐glia and microglia will help provide a novel method to modulate myelination and treat neurodegenerative disorders.

## Graphical abstract

Summary of interaction between NG2‐glia and microglia. NG2‐glia proliferation, migration, differentiation, and apoptosis are influenced by factors released from the microglia. On the other hand, NG2‐glia also regulate microglia actions.

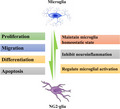

## 1 INTRODUCTION

Glia are non‐neuronal cells, also called neuroglia.^1^ Glia exist within the mammalian nervous system to provide several essential functions for neurons, such as maintaining homeostasis, support, protection, and generating myelin.^2^, ^3^ Traditionally, astrocytes, microglia, and oligodendrocytes are the three major classes of glia. In the last two decades, neuron glia antigen‐2 (NG2) glial cell has been considered as the fourth type of glial cell in the central nervous system (CNS).^4^ NG2‐glia are also called oligodendrocyte precursor cells^5^ and express NG2.^6^, ^7^, ^8^ NG2 is a chondroitin sulphate proteoglycan 4 (CSPG4), which is also expressed on infiltrating macrophages and activated microglia in various CNS insults.^9^, ^10^, ^11^ NG2, together with platelet‐derived growth factor receptor alpha (PDGFRα), is a marker of NG2‐glia.^12^, ^13^

NG2‐glia account for 5%–10% of all cells in the adult CNS.^14^ NG2‐glia are extremely heterogeneous with different characteristics and functions.^15^, ^16^ NG2‐glia are widely scattered in the CNS in late development and throughout adulthood. NG2‐glia may differentiate into neurons or astrocytes under diverse conditions, such as at embryonic stages, and under forced expression of neurogenic factors.^17^, ^18^, ^19^ Early postnatal NG2‐glia can become multipotent neurospheres and quickly differentiate into neurons, astrocytes, and oligodendrocytes in vitro.^18^ More than one‐third of the protoplasmic astrocytes are differentiated from NG2‐glia in ventral forebrain when NG2‐Cre is prenatally triggered in NG2‐glia before embryonic day 17.5.^20^ A single homeodomain transcription factor Dlx2‐transfects NG2‐glia differentiated into GABAergic inhibitory neurons.^19^ Adult NG2‐glia also differentiate into neurons in the cortical gray matter.^21^ The anti‐epileptic epigenetic regulator valproic acid accelerates the differentiation of neurons from NG2‐glia with neurogenic means in vitro.^17^

NG2‐glia are produced from neural stem cells via glial‐restricted progenitors massively in both the developing and mature CNS.^22^ NG2‐glia appear in three distinct regional waves at distinct stages in the developing murine spinal cord and forebrain.^23^, ^24^, ^25^ This is now confirmed also in the human brain.^26^, ^27^, ^28^ Moreover, NG2‐glia are homogenously distributed throughout the CNS tissue (including gray and white matter), arranged in a grid‐like manner.^29^, ^30^ NG2‐glia primarily differentiate into oligodendrocytes for myelin plasticity in the healthy CNS.^31^, ^32^ NG2‐glia proliferate and alter their morphology in response to distinct CNS insults, such as demyelinating disorders,^33^, ^34^ ischemia,^35^ stab wounds,^36^ and spinal cord injury.^33^ In the early period of multiple sclerosis, NG2‐glia maintain successful remyelination.^37^ In response to myelin damage, NG2‐glia proliferate, move to damaged areas, and differentiate into mature oligodendrocytes to form new myelin.^38^ In the development of multiple sclerosis, remyelination is continually hampered due to damaged differentiation into oligodendrocytes and inefficient proliferation and migration to myelin lesions.^39^

NG2‐glia is not merely the oligodendrocyte progenitor cell (OPC), but rather a varied and heterogeneous kind of glia with different functions in the developing and adult CNS. In the past few decades, NG2‐glia have become active participants in many aspects of brain structure and function. NG2‐glia accept synaptic input from neurons and may produce action potentials. They can function as immune cells, monitoring the environment, presenting antigens, and secreting immunomodulatory cytokines.^40^, ^41^, ^42^ In multiple sclerosis, NG2‐glia is able to generate chemokines which can recruit and activate microglia. The neuro‐inflammation leads NG2‐glia to shift toward an immunomodulatory phenotype while reducing their ability to proliferate and differentiate, thus compromising their myelin‐repair function.^43^ NG2‐glia associate closely with the blood–brain barrier and help maintain its integrity.^44^

Microglia is deemed as the tissue macrophages of the brain. Fate‐mapping analysis reveals microglia are fairly different from blood‐borne macrophages and originate from erythromyeloid precursors in the yolk sac. Brain microglia themselves can completely maintain the population and are not renewed from the blood under the normal state. But in special cases, including massive loss of microglia or autoimmune diseases, inflammatory CCR2+ monocytes generated from the definitive hematopoiesis enter the brain and become post‐inflammatory macrophages with microglia‐like characteristics.^45^, ^46^ Microglia own several functions via interacting with the other cells in the CNS, such as astrocytes, neurons, and oligodendrocytes. Microglia become a new potential therapeutic target for many neuropsychiatric disorders.^47^ Distinct microglial morphological, ultrastructural, and molecular states contribute contextually relevant functions across health and disease. Emerging knowledge on microglial heterogeneity provides an opportunity for the development of selective modulators of beneficial and/or aberrant microglial functions in a context‐dependent manner.^48^

Both the activated and resting microglia are greatly dynamic. Under normal physiological states, keeping in a functionally resting condition (M0)，microglia are ramified states with many processes and branches, extending from somata to bulbous endings. These processes constantly moving, retracting, and protruding to cover long distances and check large areas of the CNS. Because of their moving speed, microglia entirely check the brain parenchyma several times per day.^49^ Microglia activation leads to either pro‐inflammatory (M1) or anti‐inflammatory (M2) phenotypes. Generally speaking, the M1 phenotype reacts to immune stimulation by generating oxidative metabolites and the secretion of pro‐inflammatory factors, including tumor necrosis factor‐α, interleukin‐1β, and interleukin‐6, while the M2 phenotype promotes phagocytosis and improves neuronal survival with the secretion of anti‐inflammatory factors, including TGF‐β, interleukin‐4, interleukin‐10, and other neurotrophic factors.^50^, ^51^ Through both M1 and M2 microglia own phagocytic receptors, the M2 phenotype has higher phagocytic capabilities to clear dead neurons than the M1 phenotype.^52^ The M1 phenotype inaccurately engulfs normal neurons, while the M2 phenotype has neuroprotection through antioxidant functions.^53^

Sex has been gradually recognized as a vital biological variable in the treatment of neurodegenerative disorders (such as stroke) and sexually dimorphic microglia are potential targets.^54^ There are remarkabe differences between microglia and neuro‐immune signaling in the male and female brain during the whole life, and these differences may facilitate a huge difference in the process and repair of CNS damage and neurodegeneration.^55^ The molecular mechanisms resulting in microglia activation and polarization of phenotypes may be affected by sex, leading to a different response in post‐stroke between males and females.^56^ Aging is also the main factor that may have a significant influence on microglia activity. Senescence‐related changes in microglia genomic profiles and functional damages cause aging‐afforded vulnerability and poorer recovery after cerebral ischemia.^57^ Similarly, aged microglia have impaired upregulation of pro‐angiogenic genes after distal middle cerebral artery occlusion.^58^ Multiple tracers have been studied to clarify the levels of microglia activation in normal aging and in CNS diseases.^59^

Microglia play diverse neuroimmune functions in the CNS.^60^ During development, microglia help modify neural circuits through sculpting neuronal synapses and regulating the strength of synaptic transmissions.^61^ In the white matter, microglia with a distinctive amoeboid morphology intrude the corpus callosum and phagocytose NG2‐glia during early postnatal development before myelination in a CX3C chemokine receptor 1 (CX3CR1)‐dependent manner to regulate myelination of axons. Furthermore, NG2‐glia contacted or phagocytosed by microglia show no markers of cell death, indicating a new homeostatic mechanism promoting a proper NG2‐glia: axon ratio for suitable myelination. The distinctive morphology of microglia and the restricted window for phagocytosis of NG2‐glia suggest an important period for NG2‐glia engulfment, which is vital for action potential propagation during development when activity‐dependent mechanisms modulate synaptic pruning.^62^ During CNS impairment, microglia are in charge of phagocytosis and ablation of dead cells, microbes, protein aggregates, and other particulate and soluble antigens that may threaten the CNS. Moreover, microglia release several soluble factors, including cytokines, chemoattractants, and neurotropic factors, to affect numerous aspects of immune responses and tissue repair in the CNS.^63^

## 2 FUNCTION OF MICROGLIA ON NG2‐GLIA

As brain immune cells, microglia are linked to the pathogenesis of numerous brain inflammatory and neurodegenerative disorders. Microglia can self‐renew and are long lived.^64^ Regulated by local environmental signals,^65^ microglia can gain distinct activation conditions, from protective^66^ to harmful^67^ phenotypes involved in mechanisms of tissue repair and impairment. In actively demyelinating lesions, more than 80% of multiple sclerosis‐specific genes are associated with T cell‐mediated inflammation and microglia activation.^68^ Inhibiting microglia activation represses disease development,^67^ indicating a significant relationship between microglia activation and demyelination.^39^ Patterns of microglial gene expression imply advantageous microglia effects, such as engulfment of myelin debris and growth factor secretion in acute injuries and at the active border of chronic active injuries.^38^, ^69^ Without pro‐regenerative microglia, NG2‐glia differentiate at low efficiency, ultimately resulting in deficient myelin repair.^69^, ^70^ Microglia with an anti‐inflammatory phenotype increase remyelination and improve symptoms of experimental autoimmune encephalomyelitis.^71^

Interactions between microglia and myelin, the glial sheath on nerve fibers crucial for quick neural impulse transmission, are frequently investigated in the context of neurotrauma and disorder. But crosstalk between microglia and myelin under normal physiological states are largely ignored. The distinctive features of microglia evident in pathological conditions also allow microglia to modulate myelination during development and throughout adulthood. This contains engulfment of cells and myelin membrane, and the secretion of cytokines, trophic factors, and chemokines. The capability of microglia to apperceive neuronal activity and molecular characteristics of the microenvironment facilitates them to optimize myelination by impacting early oligodendrogenesis, myelination, and elimination of abnormally targeted myelin. Comprehending how microglia involve in myelination under physiological states offers a novel perspective which will raise understanding of developmental abnormalities.^72^

Microglia were previously believed to rapidly react to any pathological incidents in the brain and to help recover homeostasis; however, now this passive role of microglia in reacting to injury falls short in showing their complicated functionalities in the CNS. In fact, microglia may themselves exert neural dysfunction and neurodegeneration via loss of homeostatic and/or gain of abnormal function. Hence, microglial functions become potential targets for CNS diseases.^73^ Microglia often interact with surrounding resident cells. Immune responses are precisely modulated in the process of initiation or resolution to maintain the CNS balance in a physiological state. The switch of microglia phenotypes is determined by the disorder, its stages and severity.^74^

Decreased generation of NG2‐glia and oligodendrocytes in NG2‐glia‐specific NG2‐null (NG2‐glia‐NG2KO) mice is linked to lower myelin repair. Decreased demyelination in myeloid‐specific NG2‐null (My‐NG2KO) mice may be due to a near 70% reduction in myeloid cell migration to injury. Decreased numbers of macrophages/microglia may then cause impaired myelin repair through inhibited clearance of myelin fragments and decreased stimulatory efficacies on NG2‐glia.^75^

An extremely activated amoeboid microglia phenotype occurs during the early postnatal phases in myelinating areas of the murine CNS. These engulfed microglia subpopulations originate from early prenatal precursors and are independent of blood monocytes and cortical microglia, which already have properties of quiescent sessile microglia. Moreover, these microglia keep the population of NG2‐glia in the healthy adult CNS.^76^ Different from healthy adult and inflammation‐activated cells, neonatal microglia display a distinctive myelinogenic and neurogenic phenotype. A CD11c^+^ microglial subset, which predominates in initial myelinating regions of the developing brain, expresses genes for neuronal and glial migration, survival, and differentiation. These cells are the main source of insulin‐like growth factor 1, and their specific ablation from CD11c^+^ microglia results in the damage of initial myelination. CD11c‐targeted toxin administration causes a specific transcriptional response in neonates, different from adult microglia. CD11c^+^ microglia exist in clusters of repopulating microglia after experimental deletion and in neuro‐inflammation in adult mice, and despite some similarities, they do not recapitulate neonatal microglial features. So, the distinctive phenotype of neonatal microglia provides essential signals for myelination and neurogenesis.^77^

**FIGURE 1:**
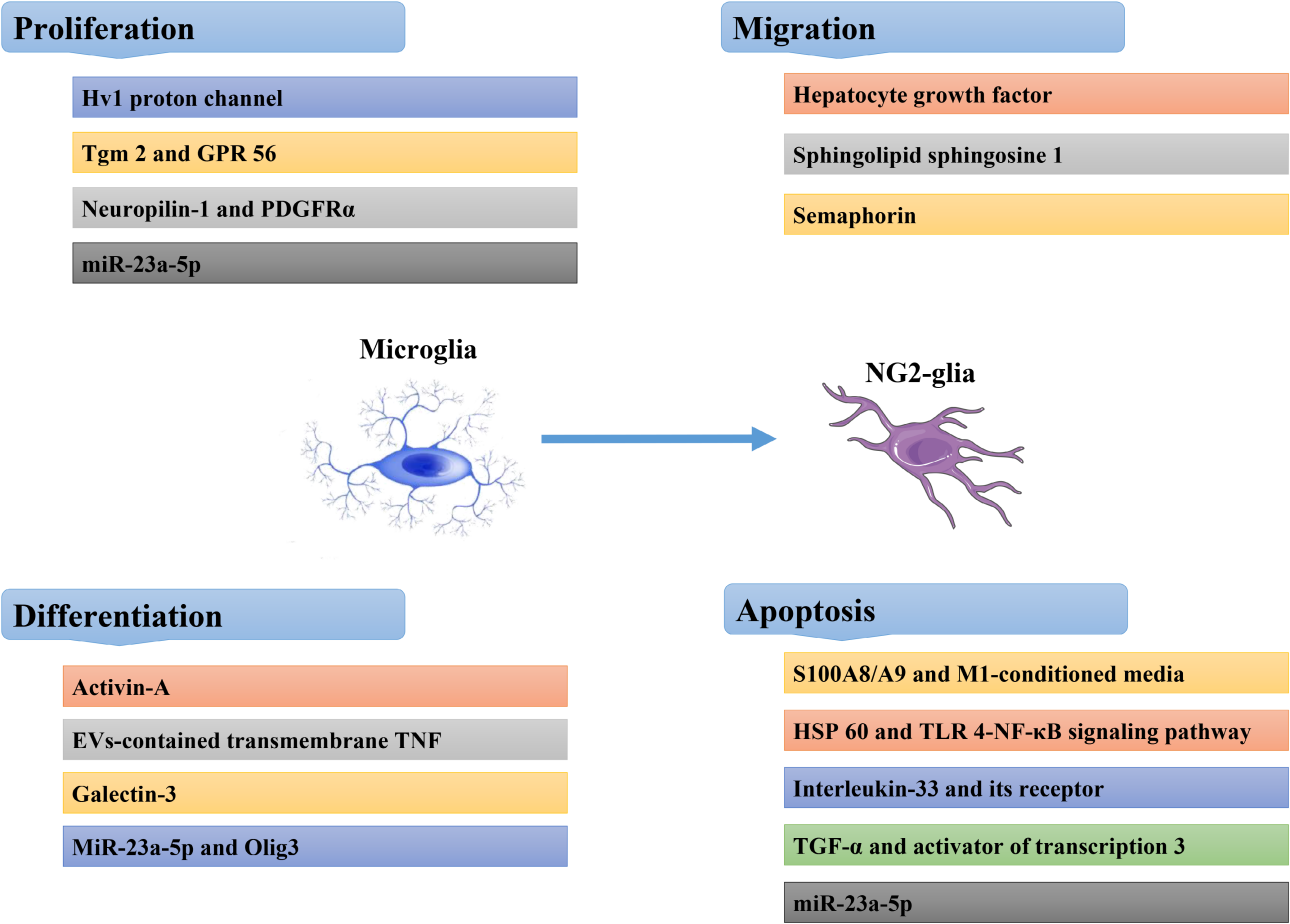
Roles of microglia on NG2‐glia in physiological and pathological states. Microglia regulate NG2‐glia proliferation through Tgm2, Hv1, neuropilin‐1 and miR‐23a‐5p; modulate migration via sphingolipid sphingosine 1 phosphate, hepatocyte growth factor, and semaphorin; improve NG2‐glia differentiation through Activin‐A, galectin‐3, EVs contain TNF and miR‐23a‐5p; and promote NG2‐glia apoptosis via the NF‐κB pathway which is related to S100A8/A9 and HSP60. Interleukin‐33, TGF‐α, and miR‐23a‐5p contribute to the survival of NG2‐glia

Activation of NG2‐glia appears to be temporally and spatially linked to the migration of activated microglia into the lesion core in spinal cord injury.^78^ NG2‐glia with different reactive morphologies emerge at the lesion edge beginning 2 days postlesion. NG2‐glia homogeneously scatter throughout the entire lesion core by 14 days after lesion initiation. Moreover, NG2‐glia numbers in the lesion border are remarkably higher than those in the microglia‐free lesion core 3 days postlesion. This raise in NG2‐glial numbers is due to increased NG2‐glia proliferation at the lesion border, as measured by the BrdU labeling test.^79^ These results suggest that unlike microglia, pre‐existing NG2‐glia survive and become reactive cells. This transformation occurs in relation to the activation and migration of microglia/macrophages in the lesion core during disease development. A tight relationship between activated microglia and NG2‐glia has previously been observed after many categories of impairment. This indicates the existence of functional and complex interplay between NG2‐glia and activated microglia/macrophages.^78^, ^79^ Activated microglia/macrophages influence the proliferation and differentiation of NG2‐glia after CNS injury.^79^ These data imply that activation of NG2‐glia may be partly caused by numerous chemokines, cytokines, and other proteins released by activated microglia/macrophages in the damaged CNS,^80^ but the precise mechanism is still unclear. The different functions of microglia on NG2‐glia are shown in Table 1 and Figure 1.

**TABLE 1:** Function of microglia on NG2‐glia

Function	Influence factor	Results	References
Proliferation	M2 microglia supernatants	Increase NG2‐glia proliferation	[81]
	M1 microglia supernatants	Inhibit NG2‐glia proliferation	[82]
	Tgm 2 and GPR 56	Microglia transglutaminase 2 drives NG2‐glia proliferation via G protein‐coupled receptor 56	[83]
	Hv1 proton channel	Microglia Hv1 inhibits NG2‐glia proliferation via reactive oxygen species	[84, 85]
	Neuropilin‐1 and PDGFRα	Microglia neuropilin‐1 trans‐actives PDGFRα on NG2‐glia and increases PDGF AA‐triggered NG2‐glia proliferation	[86]
	miR‐23a‐5p	M2 microglia‐derived EVs promote NG2‐glia proliferation via miR‐23a‐5p	[87]
Migration	Sphingolipid sphingosine 1	Microglia sphingolipid sphingosine 1 phosphate improves NG2‐glia migration	[88]
	Hepatocyte growth factor and its receptor c‐Met	Hepatocyte growth factor is secreted by microglia after treatment with prostaglandin E2 and promotes the migration of NG2‐glia	[89, 90]
	Semaphorin	Microglia semaphorin promotes NG2‐glia migration	[91, 92]
Differentiation	M1‐conditioned media	M1‐conditioned media decrease NG2‐glia differentiation	[93]
	M2‐derived activin‐A	M2‐derived activin‐A increases NG2‐glia differentiation	[69, 94]
	Galectin‐3	Microglia galectin‐3 drives NG2‐glia differentiation and results in myelin integrity and function	[95, 96]
	Extracellular vesicle‐contained transmembrane tumor necrosis factor	Microglia‐derived extracellular vesicles improve NG2‐glia differentiation	[97]
	MiR‐23a‐5p and Olig3	M2 microglia‐derived EVs' miR‐23a‐5p promotes NG2‐glia differentiation via directly targeting Olig3	[87]
Apoptosis	S100A8/A9 and M1‐conditioned media	S100A8/A9 causes the activation of microglia and increases the generation of pro‐inflammatory factors by activating the NF‐κB signaling pathway, which further deteriorates NG2‐glia injury	[98]
	Heat shock protein 60 and Toll‐like receptor 4‐NF‐κB signaling pathway	Heat shock protein 60 secreted from M1 microglia may induce NG2‐glia apoptosis by combining with Toll‐like receptor 4 on the NG2‐glia membrane to activate the Toll‐like receptor 4‐NF‐κB signaling pathway	[99]
	interleukin‐33/interleukin 1 receptor‐like 1/signal transducer and activator of transcription 6	The interleukin‐33/interleukin 1 receptor‐like 1/signal transducer and activator of transcription 6 signaling leads to an anti‐inflammatory microglia response, which preserves NG2‐glia and oligodendrocytes early after stroke	[100]
	Transforming growth factor‐α and signal transducer and activator of transcription 3	Transforming growth factor‐α protects NG2‐glia via signal transducer and activator of transcription 3 signaling	[101]
	miR‐23a‐5p	M2 microglia‐derived extracellular vesicles promote NG2‐glia survival via miR‐23a‐5p	[87]

### 2.1 Proliferation

#### 2.1.1 Microglia polarization influences NG2‐glia proliferation

Microglia can polarize to either pro‐inflammatory M1 phenotype or anti‐inflammatory/pro‐regenerative M2 phenotype in differ stages of demyelinating lesions, endogenous oligodendrogenesis is tightly linked to the dynamic transition of microglia from M1 to M2 phenotype,^81^ M2 microglia are proved to facilitate remyelination^82^ and their extracellular vesicles (EVs) mediate cellular function in cerebral lesion.^83^ M2 microglia‐derived EVs improve neurological functional recovery and white matter mend after cerebral ischemia by raising NG2‐glia survival, proliferation, and differentiation. Furthermore, the M2‐EVs' miR‐23a‐5p has a critical role in promoting NG2‐glia proliferation.^84^ Fumaric acids can shift microglia into M2 anti‐inflammatory type even under inflammatory conditions, and the supernatants of fumaric acids‐exposed microglia result in increased NG2‐glia proliferation.^85^ However, M1 microglial conditioned medium from LPS‐activated microglia attenuated primary NG2‐glia proliferation. In contrast to NG2‐glia, lipopolysaccharides‐activated M1 microglial conditioned medium enhanced the survival of mature oligodendrocytes. Further investigation suggested that tumor necrosis factor and interleukin‐6 released from M1 microglia might contribute to the inhibiting effect on NG2‐glia proliferation.^86^

#### 2.1.2 Microglial Tgm2 increases NG2‐glia proliferation

Microglia‐derived transglutaminase 2 (encoded by Tgm2) is a newly identified adhesion G protein‐coupled receptor 56(also called GPR56) ligand for NG2‐glia. Microglia‐specific knockout of Tgm2 results in decreased NG2‐glia division, fewer mature oligodendrocytes, and hypomyelination during postnatal CNS development, phenocopying the downregulation of adhesion G protein‐coupled receptor. In vitro, the activation of adhesion G protein‐coupled receptors by Tgm2 requires the extracellular matrix protein laminin to increase NG2‐glia proliferation.^87^

#### 2.1.3 Microglial Hv1 inhibits NG2‐glia proliferation via reactive oxygen species

Hv1 (encoded by the Hvcn1 gene), a newly described voltage‐gated proton channel, is ideally responsible for compensating nicotinamide adenine dinucleotide phosphate oxidase (NOX) activation by detecting both pH and voltage gradients.^88^, ^89^ However, NOX‐dependent reactive oxygen species generated in microglia are remarkably toxic to NG2‐glia. The levels of oxygen–glucose deprivation‐induced reactive oxygen species and pro‐inflammatory cytokine generation are significantly lower in Hv1‐knockout microglia than in wild type microglia. Following oxygen–glucose deprivation, NG2‐glia co‐cultured with wild type microglia exhibit increased apoptosis and decreased proliferation and maturation of NG2‐glia, but those co‐cultured with Hv1‐deficient microglia exhibit declined apoptosis and increased proliferation and differentiation of NG2‐glia. Moreover, the weakened damage and increased proliferation of NG2‐glia are linked to declines in extracellular signal‐regulated kinase 1/2 and p38 mitogen‐activated protein kinase phosphorylation. The protective functions of Hv1 knockout on NG2‐glia are because of the inhibition of reactive oxygen species and pro‐inflammatory factor generation in microglia.^90^

The Hv1 proton channel is necessary for cuprizone‐triggered microglia activation and reactive oxygen species generation. Mice lacking Hv1 show reduced reactive oxygen species production and increased proliferation of NG2‐glia and are, in part, protected from demyelination and motor dysfunction after curpizone exposure.^91^

#### 2.1.4 Microglial neuropilin‐1 promotes NG2‐glia proliferation

The type 1 integral membrane protein neuropilin‐1 is expressed on both NG2‐glia and amoeboids and activates microglia in white matter, but not in gray matter, in an activity‐ and age‐dependent manner. Microglia‐specific knockout of neuropilin‐1 impairs developmental NG2‐glia proliferation in white matter, NG2‐glia migration, and subsequent differentiation for remyelination after acute demyelination. Exogenous neuropilin‐1 increased PDGF AA‐triggered NG2‐glia proliferation and PDGFRα phosphorylation in isolated NG2‐glia, most strikingly when incubated with suboptimum levels of PDGF AA. These results reveal a mechanism of modulating oligodendrocyte lineage cell density, which implicates transactivation of PDGFRα on NG2‐glia through neuropilin‐1 expressed by near microglia.^92^

### 2.2 Migration

#### 2.2.1 Microglial sphingolipid sphingosine 1 phosphate improves NG2‐glia migration

The production of microglia EVs plays a role in the interaction between microglia and NG2‐glia, and EVs secreted from inflammatory microglia lead to the suppression of NG2‐glia differentiation into oligodendrocytes. In contrast, mesenchymal stem cells treated with EVs secreted from microglia promote remyelination. The results of the study indicate that microglia EVs become multimodal and multitarget signaling mediators and can affect both NG2‐glia and astrocytes around myelin injury, which may be a future novel method for myelin repair not only in multiple sclerosis, but also in neurological and neuropsychiatric disorders featured by demyelination. The inflammatory cargo of EVs primarily contributes to blocking NG2‐glia maturation, but surface lipids are the factors responsible for the promyelinating function of EVs. Although the category of lipids to promote NG2‐glia differentiation still needs to be further determined, sphingolipid sphingosine 1 phosphate has been identified as the crucial molecule improving NG2‐glia migration.^93^

#### 2.2.2 Microglial hepatocyte growth factor facilitates NG2‐glia migration

The Th2/3‐related cytokine transforming growth factor β (TGF‐β), which is upregulated in the remission stage of the acute experimental autoimmune encephalomyelitis, stimulates the microglia to release hepatocyte growth factor and then to facilitate NG2‐glia chemotaxis.^94^ In addition to TGF‐β, interferon‐β also facilitates hepatocyte growth factor generation by microglia. Interferon‐β is used to treat multiple sclerosis patients and favors the remission stage of the disorder. TGF‐β is dramatically increased in multiple sclerosis lesions and may have a crucial role in myelin repair.^95^ The TGF‐β induced program that causes microglia to induce NG2‐glia chemotaxis is entirely suppressed by neutralizing antibodies to the hepatocyte growth factor receptor c‐Met. This result suggests that hepatocyte growth factor is the unique NG2‐glia chemotactic factor secreted upon TGF‐β stimulation by microglia. Hepatocyte growth factor is secreted by microglia after treatment with prostaglandin E2 and promotes the migration of NG2‐glia.^96^, ^97^

#### 2.2.3 Microglial semaphorin promotes NG2‐glia migration

The existence of demyelinated plaques in the CNS is a sign of multiple sclerosis. Only some plaques remyelinate, and one of the possible reasons is the deficit of migration of NG2‐glia to the damaged regions. The guidance factors semaphorin 3A and 3F direct the migration of oligodendrocytes during development. In multiple sclerosis tissue and an experimental model of demyelination, there is a local origin of semaphorin surrounding active demyelinating lesions but not for chronic plaques. The microglia and astrocyte are the primary source of semaphorin 3A and semaphorin 3F around and within active multiple sclerosis plaques.^98^, ^99^ The loss‐ and gain‐of‐function experiments in an adult murine demyelination model illustrate that semaphorin 3A harms NG2‐glia recruitment to the demyelinated region. On the other hand, semaphorin 3F overexpression increases both NG2‐glia recruitment and remyelination rate.^100^ In focal myelinotoxic mouse model of demyelination, adding recombinant semaphorin 3A (chemorepellent) to demyelinated lesions reduces NG2‐glia recruitment and remyelination, conversely, the addition of recombinant semaphorin 3F (chemoattractant), or use of transgenic mice with downregulated semaphorin 3A expression increases NG2‐glia recruitment and remyelination.^99^

### 2.3 Differentiation

#### 2.3.1 Microglia polarization modulates NG2‐glia differentiation

In two focal demyelination models (lysophosphatidylcholine and lipopolysaccharide), specific microglia/macrophage polarization is closely associated with the demyelination–remyelination procedure, likely through regulation of cytokine components, the inflammatory niche, and the NG2‐glia response.^101^ Oligodendrocyte development depends on several extracellular signals that are mainly secreted factors from nearby neurons. Although both microglia‐ and astrocyte‐conditioned medium protect NG2‐glia against growth factor withdrawal‐induced apoptosis, astrocyte‐conditioned medium is remarkably more effective than microglia‐conditioned medium in promoting long‐term oligodendrocyte survival. On the contrary, microglia‐conditioned medium increases NG2‐glia differentiation and oligodendrocyte myelination. These distinct functions of astrocyte‐ and microglia‐conditioned medium on oligodendrocyte development are mediated by different formats of cytokine/growth factors in the conditioned medium, which are linked to diversely activated intracellular signaling pathways in NG2‐glia treated with the conditioned medium.^102^ The selected protein composition of conditioned media of microglia promotes the differentiation of NG2‐glia, with upregulated expression of PDGF, insulin‐like growth factor 1, and vascular endothelial growth factor.^102^ NG2‐glia differentiation is increased in vitro in response to M2‐conditioned media and is damaged in vivo after M2 deletion in multiple sclerosis lesions. Inhibiting M2‐derived activin‐A decreases NG2‐glia differentiation during remyelination in cerebellar slice cultures. These data suggest that M2 polarization is necessary for efficient remyelination in multiple sclerosis and confirm activin‐A as a new therapeutic target for the differentiation of NG2‐glia.^69^ However, M1 microglia restrains the differentiation of NG2‐glia. In permanent distal middle cerebral artery occlusion, Type 2 diabetes promotes a shift in the microglia phenotype toward the M1 pro‐inflammatory modality. Co‐culture experiments confirm that microglia polarization toward the pro‐inflammatory phenotype under high glucose conditions inhibits the differentiation of NG2‐glia.^103^

#### 2.3.2 Microglial galectin‐3 improves NG2‐glia differentiation

Galectin‐3, a β‐galactoside‐binding lectin, has a critical role in inflammatory and neurodegenerative disorders. During cuprizone‐induced mice demyelination, galectin‐3 is upregulated in microglia and functions as a vital regulator of microglia activation and phenotype, initiating the beginning of remyelination. However, galectin‐3 deficiency leads to the decrease in the differentiation of NG2‐glia.^104^ Similarly, a previous study has demonstrated that galectin‐3 drives NG2‐glia differentiation and results in myelin integrity and function.^105^

#### 2.3.3 Microglia‐derived EVs improve NG2‐glia differentiation

After stroke, intracerebral infusion of regenerative microglia‐secreted EVs recovers protective functions of microglia, restricting their senescence during the post‐stroke stage, and augments the maturation of NG2‐glia at damaged borders, leading to improved neurological functions. In vitro studies indicate that EVs‐contained transmembrane tumor necrosis factor has the pro‐differentiating effects on NG2‐glia.^106^ Further research found that M2 microglia‐derived EVs' miR‐23a‐5p could promote NG2‐glia differentiation possibly via directly targeting Olig3.^84^

### 2.4 Apoptosis

#### 2.4.1 M1 Microglia promote NG2‐glia apoptosis

NG2‐glia survival is influenced not only via a direct response to endotoxins but also by pro‐inflammatory cytokines, such as tumor necrosis factor and interleukin‐6, secreted from endotoxin‐activated microglia.^86^ S100A8/A9, a heterodimer complex made up of calcium‐binding proteins S100A8 and S100A9, is markedly increased in the blood of multiple sclerosis patients.^107^ Both the conditioned medium of S100A8/A9‐activated BV2 cells, a microglia cell line, and S100A8/A9 contribute to NG2‐glia apoptosis, which is more significant after treatment with the conditioned medium. S100A8/A9 causes the activation of BV2 cells and increases the generation of pro‐inflammatory factors by activating the NF‐κB signaling pathway, which further deteriorates NG2‐glia injury.^108^ Similarly, M1 microglia‐conditioned medium exacerbates oxygen glucose deprivation‐induced NG2‐glia death.^109^

#### 2.4.2 Microglia activation triggers NG2‐glia apoptosis via HSP 60

Reactive microglia exist in the lesions of myelin‐related white matter diseases that lead to NG2‐glial damage.^110^ Heat shock protein 60(HSP60) secreted from M1 microglia may induce NG2‐glia apoptosis by combining with Toll‐like receptor 4 (TLR4) on the NG2‐glia membrane to activate the TLR4‐NF‐κB signaling pathway.^111^

#### 2.4.3 Microglia protect NG2‐glia from apoptosis via TGF‐α

Interleukin‐33 and its receptor (interleukin‐1 receptor‐like 1) have vital roles in normal CNS and neurological diseases. Microglia are the chief targets of interleukin‐33 in the CNS. Interleukin‐33/interleukin 1 receptor‐like 1/signal transducer and activator of transcription 6 signaling results in an anti‐inflammatory microglia reaction, which protects NG2‐glia and oligodendrocytes early after stroke and keeps white matter integrity in a long run.^112^ TGF‐α is largely expressed in the CNS. Microglia‐derived TGF‐α owns anti‐apoptotic functions on NG2‐glia and oligodendrocytes. Moreover, TGF‐α may raise the pool of surviving NG2‐glia, which are available for differentiation into myelin‐generating oligodendrocytes to performing endogenous myelin mend. Signal transducer and activator of transcription 3 signaling pathway is involved in the functions of TGF‐α on NG2‐glia.^113^

## 3 FUNCTION OF NG2‐GLIA ON MICROGLIA

**FIGURE 2:**
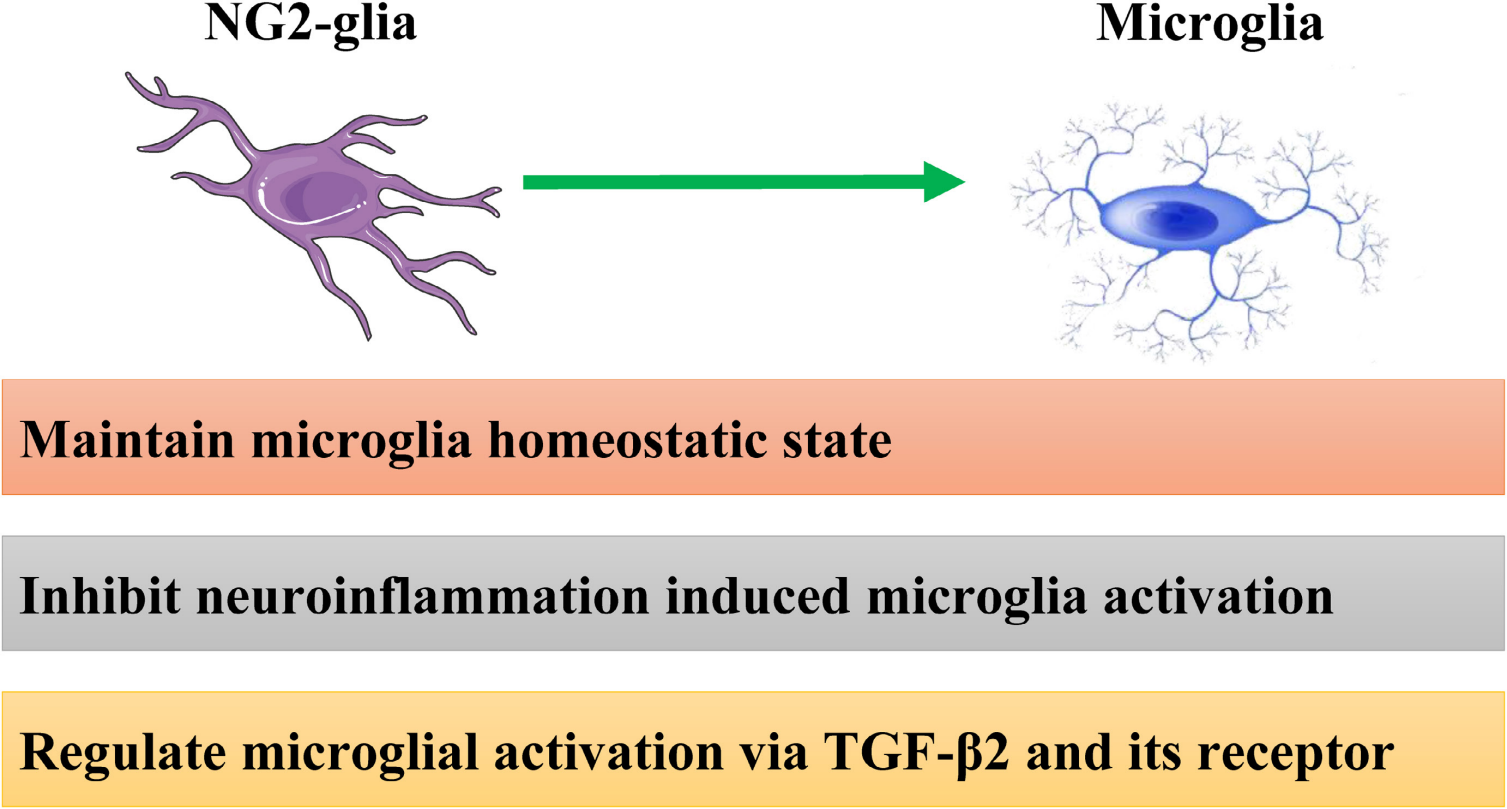
Roles of NG2‐glia on microglia in physiological and pathological states. NG2‐glia maintain a microglia homeostatic state, suppress neuroinflammation induced by microglia activation, and modulate microglia activation via TGF‐β2 and its receptor

NG2‐glia also regulate microglia actions. The different functions of NG2‐glia on microglia are shown in Table 2 and Figure 2.

**TABLE 2:** Function of NG2‐glia on microglia

Function	Influence factor	Results	References
Maintain microglia homeostasis	The deletion of NG2‐glia	Deletion of NG2‐glia interferes microglia homeostatic state	[114]
Inhibit neuroinflammation	Hepatocyte growth factor	Hepatocyte growth factor sustains the survival of hippocampal neurons	[115]
Regulate microglia activation	TGF‐β2 and its receptor	NG2‐glia regulate microglia activation via TGF‐β2 and its receptor	

### 3.1 NG2‐glia maintain microglia homeostasis

Microglia have crucial roles in the health and impairment of the CNS. An imbalance in microglia homeostasis is a primary characteristic of CNS aging and neurodegeneration.^114^ Delete NG2‐glia with high efficiency and selection in cultured brain slices reveals that depletion of NG2‐glia abrogates the homeostatic microglia signature, but does not impact disease‐associated microglia properties. The deletion of NG2‐glia interrupts microglia homeostatic state without impairing microglia numbers. The deletion of NG2‐glia interferes microglia homeostatic state without triggering inflammatory responses in the adult mouse brain.^115^ These results demonstrate that NG2‐glia have a vital effect on microglia cellular states, which are associated with brain aging and neurodegenerative diseases.

### 3.2 NG2‐glia inhibit neuro‐inflammation induced by microglia activation

The transgenic rat expressing herpes simplex virus thymidine kinase under the control of the promoter for NG2 (NG2‐herpes simplex virus thymidine kinase Tg rats) can selectively ablate NG2‐glia in the adult CNS. The herpes simplex virus thymidine kinase/ganciclovir transgenic rats are shown apoptotic neuron and induced excessive neuroinflammation in hippocampus. Further study finds that NG2‐glia deletion‐induced neuronal apoptosis in hippocampus may be mediated by the interleukin‐1β pro‐inflammatory pathway in activated microglia, and NG2‐glia‐generated hepatocyte growth factor is demanded for the existence of hippocampal neurons. These data indicate that NG2‐glia own the potential to inhibit neuroinflammation and sustain the survival of hippocampal neurons via the hepatocyte growth factor.^116^

### 3.3 NG2‐glia regulate microglia activation via TGF‐β2 and its receptor

NG2‐glia are necessary to maintain immune homeostasis in the CNS through TGF‐β2‐TGF‐β type II receptor‐CX3C chemokine receptor 1 signaling, which inhibits microglia activation. Mice lacking NG2‐glia show a significant downregulated expression of microglia‐specific signature genes and an obvious inflammatory response in the CNS when treated with endotoxin lipopolysaccharides.^117^ NG2‐glia‐generated TGF‐β2 and its receptor TGF‐β type II receptor in microglia are critical modulators of the CX3C chemokine receptor 1‐regulated immune response. In addition, deletion of NG2‐glia results in neuroinflammation and nigral dopaminergic neuron depletion in a neurotoxin 1‐methyl‐4‐phenyl‐1,2,3,6‐tetrahydropyridine‐induced mouse Parkinson's disease model.^118^

## 4 CONCLUSION

The existence and functional responses of NG2‐glia are positively or negatively regulated by immune‐induced results.^119^ As immune cells in the CNS, microglia are extremely reactive and are activated by any alterations in the CNS,^49^ and protect against different pathogenic factors.^118^, ^120^ Microglia regulate NG2‐glia responses during myelination and remyelination, mediating microglia‐expressed transglutaminase‐2, hepatocyte growth factor, galectin‐3, and phagocytosis of debris and dead cells. Remyelination after myelin impairment in the CNS is dependent on NG2‐glia migrating into damaged regions, differentiating into mature oligodendrocytes and myelinating the axons. After myelin lesion, on one hand, microglia perform a conducive environment for the naturally occurring remyelination via cleaning the myelin debris.^121^ On the other hand, microglia increase NG2‐glia survival proliferation, migration, differentiation, and maturation though secreting regenerative factors such as TGF‐α,^113^ galectin‐3,^104^ activin‐A,^69^ and miR‐23a‐5p.^84^ Microglia regulate NG2‐glia function via EVs and M2‐EVs potentially ameliorate demyelinating disorders, such as ischemic stroke. M1 microglia secrete pro‐inflammatory factors to deteriorate NG2‐glia.^122^

Beyond their contribution to myelination, NG2‐glia own a vital role in keeping the normal structure and function of blood–brain barrier.^123^ NG2‐glia could be novel therapeutic targets in cerebrovascular disorders, especially in the context of vascular cognitive dysfunction in an aging CNS.^124^ NG2‐glia also have an important role to maintain energy homeostasis.^125^ In addition, NG2‐glia can mediate neuroinflammation through TGF‐β2‐TGF‐β type II receptor‐CX3C chemokine receptor 1 signaling to inhibit microglia activation.^117^ More studies are still needed to reveal the modulation, mechanisms of molecular and signaling pathways, and consequences of dysfunction of interaction between NG2‐glia and microglia.

## AUTHOR CONTRIBUTIONS

Zuo Zhang and Xiaolong Li wrote the manuscript. Hongli Zhou contributed to the preparation of the figures. Zuo Zhang and Jiyin Zhou edited and revised the manuscript. All authors read and approved the final manuscript.

## FUNDING INFORMATION

The National Natural Science Foundation of China (No. 81770806), the Natural Science Foundation of Chongqing (cstc2021jcyj‐msxmX0249), and Special Project for Enhancing Science and Technology Innovation Ability of Army Medical University (No. 2019XYY16) supported this work.

## CONFLICT OF INTEREST

The authors declare that the research was conducted in the absence of any commercial or financial relationships that could be construed as a potential conflict of interest.

## Data Availability

Data sharing is not applicable to this article as no new data were created or analyzed in this study.
